# Adiponectin Expression and Genotypes in Italian People with Severe Obesity Undergone a Hypocaloric Diet and Physical Exercise Program

**DOI:** 10.3390/nu11092195

**Published:** 2019-09-12

**Authors:** Graziamaria Corbi, Rita Polito, Maria Ludovica Monaco, Francesco Cacciatore, Michelina Scioli, Nicola Ferrara, Aurora Daniele, Ersilia Nigro

**Affiliations:** 1 Dipartimento di Medicina e Scienze della Salute, Università del Molise, 86100 Campobasso, Italy; 2 Dipartimento di Scienze e Tecnologie Ambientali, Biologiche, Farmaceutiche, Università della Campania “Luigi Vanvitelli”, 81100 Caserta, Italy; 3 CEINGE-Biotecnologie Avanzate, 80131 Napoli, Italy; 4 Dipartimento di scienze mediche traslazionali, Università di Napoli “Federico II”, 80131 Napoli, Italy; 5 Laboratorio della valutazione della complessità clinica, Istituti Clinici Scientifici Maugeri Spa SB, 82037 Telese, Italy

**Keywords:** obesity, adiponectin, *ADIPOQ* polymorphisms, weight loss, physical exercise

## Abstract

Adiponectin exerts positive effects on metabolic and inflammatory processes. Adiponectin levels and some single-nucleotide polymorphisms (SNPs) seem to be associated with obesity. Here, we investigated the effects of a 4-week Hypocaloric diet and Physical exercise Program (HPP) on 268 young people with severe obesity. We evaluated the relationship between adiponectin levels and anthropometric and biochemical parameters, at baseline and after a 4-week HPP. Finally, we investigated some adiponectin gene variants and their correlation to biochemical parameters. Adiponectin levels were statistically lower in people with severe obesity than in controls. At the end of the HPP, all the people with severe obesity showed a Body Mass Index (BMI) reduction with a statistically significant increase in adiponectin levels. Genotyping, the adiponectin gene demonstrated a significant difference in 3 polymorphisms within the people with severe obesity. Besides, c.11377C>G and c.11391G>A homozygous subjects experienced more advantages by HPP. Furthermore, c.268G>A heterozygous subjects showed an enhancement in lipid profile as well in adiponectin levels. The best predictor of the changes in adiponectin levels was represented by the c.268G>A WT allele. Our study confirmed that a 4-weeks HPP in people with severe obesity results in metabolic amelioration associated with a significant increase of adiponectin levels. Importantly, we found that a specific genetic background in the *ADIPOQ* gene can predispose toward a more significant weight loss.

## 1. Introduction

Obesity is a growing public health problem worldwide, representing a major risk factor for different metabolic conditions such as type 2 diabetes, atherosclerosis, and cardiovascular diseases and also for several cancers [1,2,3,4]. The underlying molecular mechanisms linking obesity to the development of additional metabolic disorders are not completely understood, but the chronic low-grade inflammation and the deregulated secretion of adipokines and other cytokines seem to be involved [4]. One of the obesity-associated hormones produced by the adipocytes is the adiponectin, whose serum levels are inversely related to body mass index (BMI) and insulin resistance [5]. Different experimental evidence showed that increased adiponectin expression is associated with raised insulin sensitivity [6,7]. The human adiponectin gene (*ADIPOQ*) consists of three exons, and it maps on chromosome 3q27, where genome-wide scans have revealed a locus susceptible for type 2 diabetes [8]. Several epidemiologic studies also reported that single-nucleotide polymorphisms (SNPs) and some haplotypes present in *ADIPOQ*, included in the promoter region, are associated with obesity and related disorders [8]. The *ADIPOQ* gene encodes a 244 amino acid monomer with a molecular weight of approximately 26 kDa. Adiponectin accounts for up to 0.05% of the total serum proteins [5] in circulation, where it can be found in three oligomeric isoforms, with different molecular weight: trimers of Low Molecular Weight (LMW), hexamers of Medium Molecular Weight (MMW), and multimers of High Molecular Weight (HMW) [9]. These latter have been correlated to the most relevant biological activities of adiponectin [10].

Adiponectin exerts many positive effects on several metabolic processes, so to be regarded as an insulin sensitizer, anti-diabetic and anti-inflammatory molecule [11]. To support this evidence, several in vivo and in vitro studies have demonstrated positive effects of adiponectin on different metabolic alterations mainly through the binding to its receptors, AdipoR1, AdipoR2 and T-cadherin [12]. Adiponectin serum levels decrease in metabolic conditions as obesity and related disorders, and several types of cancer [13,14]. Also, epidemiologic studies assessed that some *ADIPOQ* SNPs and some haplotypes are associated with obesity, cardiovascular diseases and lung cancer [15,16,17]. Furthermore, few causal SNPs have been associated with hypoadiponectinemia impaired adiponectin multimerization, and T2D [18].

Physical exercise is recognized as one of the most effective tools in the prevention and the therapy of metabolic diseases and cancer, thanks to its several positive effects on body composition, insulin sensitivity, blood glucose, and lipid levels [19,20,21,22]. In particular, physical exercise seems to exert its beneficial activities through the secretion of different hormones/cytokines involved in many pathophysiological processes [23,24]. However, the effects of physical exercise on serum adiponectin levels are controversial, depending on the exercise type, the intensity of the training and the study population; some studies, performed on patients with obesity, diabetes and at high risk for myocardial infarction, showed a positive correlation between adiponectin levels and exercise [24], while others demonstrated that basal adiponectin concentrations do not change after long-term exercise [24,25,26]. Furthermore, only a few studies examined the effects of exercise on the HMW oligomers [24,27].

In the present study, we investigated the effects of a 4-week Hypocaloric diet plus Physical exercise Program (HPP) in young people with severe obesity. In particular, we evaluated the association between circulating levels of Adiponectin and anthropometric and biochemical parameters in the patients before the HPP; then we looked at the variations in biochemical parameters after the weight loss with a particular focus on adiponectin levels. Then, we investigated (by genotyping) the entire *ADIPOQ* gene in people with severe obesity, to determine the potential presence of genetic variants (SNPs) and/or mutations, and their correlation to biochemical variations.

## 2. Materials and Methods

### 2.1. Subjects and Sampling 

The study population consisted of 268 unrelated people with severe obesity (87 [32.5%] males; mean age 42.28 ± 13.81; mean BMI: 48.90 ± 7.60 kg/m^2^), recruited from the Foundation “Salvatore Maugeri” Telese, Italy. Exclusion criteria were: type 2 diabetes mellitus, positive family history of metabolic diseases. 

One hundred and fifty healthy age-matched volunteers (49 males [32.7%]; mean age 40.84 ± 9.40; mean BMI: 23.46 ± 2.89 kg/m^2^) were recruited from the Federico II University Hospital staff as controls. All participants were Caucasians. The study was approved by the Ethics Committee of Faculty of Medicine and conducted according to the ethical principles of the Declaration of Helsinki. Informed consent was obtained from all participants. Blood samples were collected after a 12-h overnight fasting period and centrifuged to collect serum. Serum aliquots were immediately frozen in liquid nitrogen and stored at −80 °C.

### 2.2. Hypocaloric Diet Plus Physical Exercise Program (HPP)

People with severe obesity were hospitalized and therefore meals were provided to the participants, ensuring the adherence to the diet. Meals provided an average of 1200 kcal/day with a balanced diet abundant in fibre. All the people with severe obesity performed endurance exercises about 60 min to six times per week. The exercise regimen combined 40 min of treadmill walking and 20 min of cycle ergometer. The exercise program was performed for 4 weeks. Since the percentage of heart rate reserve is considered to be equivalent to % of maximal oxygen consumption (% VO2max) for exercise prescription purposes, the exercise intensity was set at the 60–70% heart rate reserve. People with severe obesity were analysed before (T0) and at the end of a 4-week HPP (T1).

### 2.3. Anthropometric and Biochemical Measurements

Table 1 shows the anthropometric and biochemical parameters of the study population. BMI was calculated as previously reported [28]; the percentage of the excess weight loss (% EWL) as the weight loss (kg) after the exercise (T1) divided by the weight (kg) before the physical exercise (T0), per 100. Systolic and diastolic blood pressures measurements were performed at least after 10 min of sitting rest. In all the participants, total cholesterol, triglycerides, glucose, High-Density Lipoprotein (HDL), Low-Density Lipoprotein (LDL) and insulin levels were assessed. Insulin resistance was estimated according to the homeostasis model assessment (HOMA), calculated with the formula: (fasting glucose x fasting insulin)/22.5, and patients were considered insulin-resistant when HOMA was ≥2.6. Furthermore, the diagnosis of metabolic syndrome was made based on the presence of at least 3 out of the 5 risk factors considered by the ATP III criteria [29].

In all the participants, the total serum adiponectin concentration was measured in triplicate by an enzyme-linked immunosorbent assay (ELISA) using a polyclonal antibody produced in-house versus a human adiponectin amino acid fragment (H2N-ETTTQGPGVLLPLPKG-COOH) as previously described [28].

### 2.4. DNA Extraction and Screening of the ADIPOQ Gene

Genomic DNA was extracted from peripheral blood leukocytes using a standard salting-out/ethanol precipitation. The exon regions, the exon-intron boundaries and the promoter region of the *ADIPOQ* gene (GeneBank accession no: NM004797) were amplified with in-house primer sets using the PCR protocol as previously described [30]. The primer sequences were as follows: 5′GCTCTGTGTGGACTGTGGAG’3 and 5′CCACACCACTCCAGGAACTT’3 for promoter; 5′CAAGGCCTGGAAACACAAGT’3 and 5′CACCTGTATCCACTCCCACA’3 for exon 1; 5′TCTCTCCATGGCTGACAGTG’3 and 5′AGCTTTGCTTTCTCCCTGTG’3 for exon 2; 5′GGAGCCACAGGGATGGTAAT’3 and 5′ATTGACTTTGGGGCTGTTTG’3 for exon 3. The PCR products were electrophoresed on a 1% agarose gel and the analysis of the sequence was performed on both strands using an automated procedure with the 3100 Genetic Analyzer (Applied Biosystem, Foster City, CA, USA). PCR fragments were sequenced using the same primers used for PCR amplification.

### 2.5. Western Blotting Analysis

Serum samples from all participants were quantified for total proteins by Bradford’s method (Bio-Rad, Hercules, CA, USA) and 10 µg of total proteins were treated with 1 × Laemmli buffer, heated at 95 °C for 10 min and loaded on 10% SDS-PAGE gel as previously described [28]. The blots were developed by ECL (Amersham Biosciences, Piscataway, NJ, USA) with the use of Kodak BioMax Light film, digitalized with a scanner (1200 dpi) and analyzed by densitometry with the ImageJ software (1.51i, Wayne Rasband (NIH), Rockville, MD, United States. http://rsbweb.nih.gov.ij/). All experiments were performed in triplicate. 

### 2.6. Statistical Analysis

Data were analyzed using the SPSS (v 23.0, Chicago, IL, USA) Software package (SPSS. Inc. Chicago, IL, USA). The Shapiro-Wilk test was used to assess the normal distribution of data. Differences between multiple groups were evaluated by analysis of variance (ANOVA) with the Bonferroni post hoc test and are presented as the means ± SD. The χ^2^ test was used to compare categorical variables. The differences between baseline and post-HPP were expressed as Delta (Δ) values. 

A multiple linear or logistic regression analysis was used to model the relationship between variables. To explore the correlation between variables, Spearman correlation r was used. The statistical significance was established at *p* < 05.

## 3. Results

### 3.1. A Hypocaloric Diet Plus Physical Exercise Program Ameliorates Biochemical Parameters of People with Severe Obesity and Increase Acpr30 Levels

The anthropometric and biochemical characteristics of the people with severe obesity and the age and gender-matched control group are reported in Table 1. Controls and people with severe obesity showed statistically significant differences for the following parameters: weight, BMI, glucose, total cholesterol, HDL, LDL, triglycerides, and fibrinogen (Table 1). The analysis of the total adiponectin levels exhibited a statistically lower concentration in the people with severe obesity than in controls (24.07 ± 6.67 vs 29.89 ± 9.8 μg/mL. *p* < 0001). 

Table 2 reports the comparison of the anthropometric and biochemical characteristics of the people with severe obesity at the baseline (T0) and at the end of the 4-week HPP (T1). All patients showed a significant body weight loss and BMI reduction compared to baseline (T0) (*p* < 0001). Also, the following parameters significantly ameliorated at T1 in respect to T0: glucose and 2-h glucose (but not HOMA index neither fasting insulin), total cholesterol, HDL, LDL, triglycerides, fibrinogen, VES and CRP (Table2). It is noteworthy that the LDL-cholesterol reached levels similar to the controls in the range of normality. At the end of the HPP, the metabolic and inflammatory profile of the people with severe obesity also significantly ameliorated.

The comparison analysis between T0 and T1 showed a significant increase in the total adiponectin levels (*p* < 0001) in the people with severe obesity undergone the HPP. 

### 3.2. High-Molecular Weight Oligomers of Adiponectin Increase at the End of a 4-Week HPP

**Figure 1:**
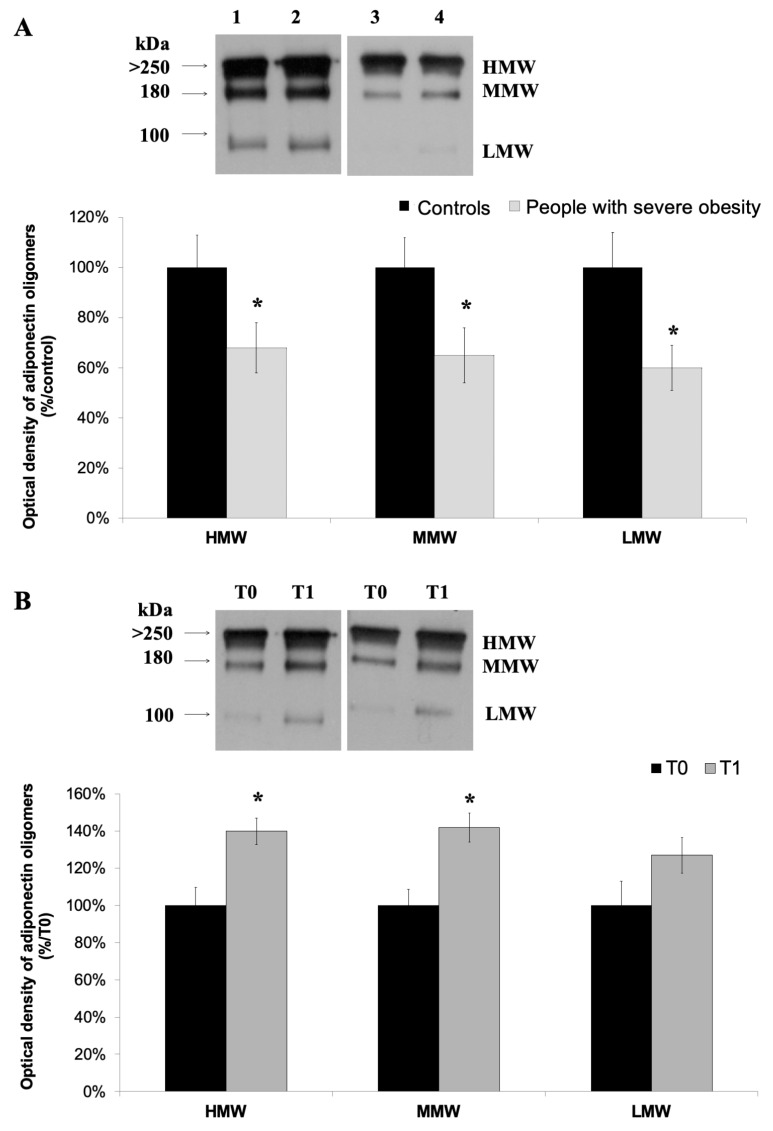
Western blotting of adiponectin and its oligomerization state in the serum of controls and people with severe obesity (**A**) at baseline, and in people with severe obesity at baseline and the end of a 4-week Hypocaloric diet and Physical exercise Program (HPP) (**B**). (**A**) Representative blot images showing different adiponectin oligomers (HMW, MMW, and LMW) in controls (lanes 1–2) and people with severe obesity (lanes 3–4). (**B**) Representative blots images of HMW, MMW, and LMW oligomers in two people with severe obesity, at T0 and T1. The graphic representation of the pixel analysis was performed using the ImageJ software, and the values are reported as mean +/- S.D. of two independent experiments performed in duplicate. The statistical significance was established at * *p* < 0.5.

To investigate the distribution of adiponectin oligomers, Western blotting was performed, in native conditions, on sera of controls and people with severe obesity: three bands corresponding to HMW (≥250 kDa), MMW (~180 kDa) and LMW (~90 kDa) oligomers were observed in both controls (Figure 1A, lanes 1–2) and people with severe obesity (Figure 1B, lanes 3–4). The densitometry evaluation of the adiponectin pattern showed a higher expression of all oligomers in controls compared with people with severe obesity (Figure 1A, *p* ≤.05). On sera of the people with severe obesity undergone HPP, the adiponectin oligomeric distribution revealed that the levels of HMW, MMW, and LMW oligomers were higher in T1 than in T0 (Figure 1B; *p* < 05). The densitometry evaluation of the adiponectin oligomers still showed a higher expression of the three oligomers at T1 compared at T0, with a significant increase of HMW and MMW; the LMW species also increased, but this raise did not reach a statistical significance (*p* = 08).

### 3.3. ADIPOQ Polymorphisms are Related to the Effects of a 4-week HPP

We analyzed the genotype of the *ADIPOQ* gene in controls and people with severe obesity investigating their distribution. Firstly, Hardy-Weinberg equilibrium about the genotype frequencies of polymorphisms was assessed using a chi-square goodness-of-fit test (*p* > 05) (supplementary Table S2).

We found a significant difference in the distribution of 3 polymorphisms between the two groups, rs17300539 c.11391G>A, rs60806105 c.11156insCA, and rs1501299 c.214+62G>T (Table 3). The first and the second are localized in the promoter region, while the third is found in the intron-2 region (supplementary Figure S1). For all the three polymorphisms, the frequency of the heterozygous subjects was higher among people with severe obesity respect to control participants (*p* < 012, *p* < 018 and *p* < 0001, respectively).

To analyse whether the considered adiponectin polymorphisms were associated to and, therefore, could control and modify the response to the HPP in people with severe obesity, we investigated the relationship between the adiponectin polymorphisms and the changes in anthropometric and biochemical parameters after HPP (expressed as Δ value, calculated as differences between T1 and T0).

**Figure 2:**
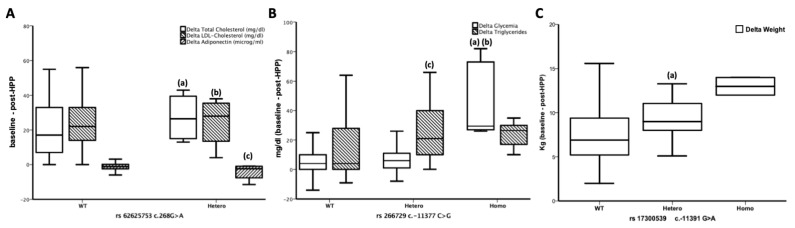
(**A**) Differences in Δ Total Cholesterol, Δ LDL Cholesterol and Δ adiponectin among the three groups based on rs62625753 polymorphism. At the end of a 4-weeks HPP in respect to baseline, the Heterozygous showed a significantly greater reduction in (a) Total Cholesterol (*p* = 0.010), and in (b) LDL Cholesterol (*p* = 0.036) than WT, with a significant increase in (c) adiponectin levels (*p* = 0.005). (**B**) Differences in Δ Glycemia and Triglycerides among the three groups based on rs266729c polymorphism. At the end of 4-weeks HPP in respect to baseline, the Homozygous showed a significantly greater reduction in Glycemia levels than both (a) WT (*p* < 0.0001) and (b) Heterozygous (*p* < 0.0001). On regard to Triglycerides levels, the Heterozygous showed a significantly greater reduction than (c) WT (*p* < 0.0001). (**C**) Differences in Δ Weight among the three groups based on rs17300539 polymorphism. At the end of a 4-weeks HPP compared to baseline, the Heterozygous showed a significantly greater reduction (*p* = 029) in weight than (a) WT. Although an increasing trend was found in Homozygous no significant results were found because of the small size of this group (*n* = 2 patients).

Homozygous subjects for the polymorphism rs266729 c.11377C>G experienced more advantages by HPP, as showed by higher Δ glucose and Δ triglycerides (both *p* < 0001, Table 4). Similarly, subjects homozygous for the polymorphism rs17300539 c.11391G>A showed a higher Δ weight. Then, the heterozygous for rs62625753 c.268G>A presented enhancement in lipid profile, as well in adiponectin levels (Table 4). Figure 2 summarizes these results.

### 3.4. rs62625753 Predicts Δ Adiponectin

A multivariate analysis was performed to test the association between the adiponectin levels, the polymorphisms in the *ADIPOQ* gene and the other variables (age, gender, etc.). Only the variables reaching a *p*-value <.10, at the univariate analysis, were introduced in the multivariate analysis model. 

The multivariate linear regression analysis, performed on the people with severe obesity undergone HPP, showed that the best predictor of Δ adiponectin, used as the dependent variable, was represented by the WT allele of rs62625753 c.268G>A (β = −3.290; 95% CI −5.872 −0.709. *p* = 0.013). Similarly, by using the rs62625753 c.268G>A polymorphism as the dependent parameter in a multivariate logistic regression analysis, the best predictor of WT allele was represented by the Δ adiponectin (β = −0.642; 95% CI −0.446 −0.924. *p* = 017). In our population, for the same allele, no homozygous subjects were found.

## 4. Discussion

Life-style modifications have triggered the epidemic of obesity, which is associated with an increased risk of many comorbid conditions like diabetes, metabolic syndrome, cancer and lung diseases. Obesity is characterized by a low-grade chronic inflammatory status of the adipose tissue, with a deregulation in the production and secretion of adipokines. Among the others, the adiponectin is the most abundantly molecule secreted in serum. Adiponectin has been largely studied in obesity and related diseases [31,32,33,34], and well-known are its insulin-sensitizing, anti-atherogenic, and anti-inflammatory properties. Also, HMW adiponectin oligomers have key roles in energy metabolism and are involved in obesity associated metabolic disorders [5].

Here, in a large cohort of young patients affected by severe obesity we investigated the effects of a 4-week program consisting in the association of a Hypocaloric diet plus Physical exercise evaluating, in association with the genetic background, the correlation between circulating levels of adiponectin and anthropometric and biochemical parameters at baseline; then, we looked at the variations in biochemical parameters (Δ values) at the end of the HPP with a particular focus on adiponectin. 

At baseline, our cohort of people with severe obesity demonstrated a statistically lower concentration of total adiponectin than controls. Furthermore, by the analysis of the adiponectin oligomeric status, all three adiponectin oligomers were significantly less expressed in people with obesity. These data are congruous with most of the studies in literature that largely agree on reporting adiponectin levels inversely correlated to BMI [5].

Successively, we analysed the genotype of the *ADIPOQ* gene in both normal weight and people with severe obesity, finding a significant difference in the distribution of 3 polymorphisms within the population with obesity (rs17300539 c.11391G>A, rs60806105 c.11156insCA, rs1501299 c.214+62G>T). Previously, the rs17300539 SNP in the promoter region of the *ADIPOQ* gene was shown to be strongly associated with plasma adiponectin levels [35]. Gong et al., according to our data, reported that the rs17300539 A allele is a risk factor for type 2 diabetes even if only in European Caucasians [36]. Similarly, the rs1501299 SNP was previously found to be associated with adiponectin level in obese Japanese subjects, therefore supporting our findings [37]. 

The rs60806105 SNP was previously associated with lower obesity levels, in particular with reduced BMI, waist circumference, subcutaneous adipose tissue and visceral adipose tissue. Thus, the polymorphism was reported to have a protective effect against obesity [38].

Other population studies have reported that a large number of SNPs independently contribute to the variation of adiponectin levels [39,40,41]. Nine genotyped tagSNPs in *ADIPOQ* have been associated with serum adiponectin after adjustment for age, gender and BMI, including rs10937273, rs12637534, rs1648707, rs16861209, rs822395, rs17366568, rs3774261, rs6444175 and rs17373414. A multi-SNP genotypic risk score for some *ADIPOQ* alleles revealed an association with 3 independent SNPs, rs12637534, rs16861209, rs17366568 and type 2 diabetes, after adjusting for adiponectin levels [30].

One possible explanation for the observed discrepancies may be due to synthetic associations i.e., the fact that population-specific rare variants are in partial linkage disequilibrium with the common ones. Also, gene-gene and gene-environment interactions may explain the remaining heritability.

It is to notice that we previously analyzed the above-mentioned SNPs in a cohort of professional athletes, failing to find any difference with sedentary controls or any association with biochemical parameters [42]. These observations might suggest that a relevant genetic background is detectable only in metabolic morbid conditions.

Weight loss is a complex trait that depends on many environmental, behavioural and genetic influences. In our study, after a 4-week HPP (T1), all the people with severe obesity showed a statistically significant body weight loss and BMI reduction compared to baseline (T0), with a significant increase in total adiponectin levels, suggesting a positive effect of the combined association of a structured diet and physical exercise on this parameter. Interestingly, we found that the increase in adiponectin levels was mainly due to HMW and MMW oligomers, the most biologically relevant adiponectin oligomers form. Our data are according to published evidence, reporting an increase in HMW and MMW oligomers after weight loss [43,44]. The involvement of specific oligomers suggests that the 4-week HPP produces functional effects on the adipose tissue that increases circulating levels of the most biologically relevant adiponectin oligomers. Accordingly, in literature, several studies found that after weight loss, reached by surgery or diet or physical exercise, adiponectin serum levels increase both in adult or pediatric obese patients [33]. On the other hand, many studies also described no changes in adiponectin levels in case of normal subjects undergone physical exercise or hypocaloric diet, or in case of modest weight loss. In particular, Madsen et al. suggested that a weight loss larger than 10% is needed to obtain an increase in adiponectin serum levels [34]. 

Then, in our cohort of people with severe obesity, we analyzed the possible influence of the genetic background in the *ADIPOQ* gene on the weight loss, as well as on the amelioration of the biochemical profile. 

In particular, after a 4-week HPP in respect to baseline, we found that the Heterozygous for the rs62625753 polymorphism showed a significantly greater reduction in Total Cholesterol, and in LDL Cholesterol than WT, with a significant increase in adiponectin levels. Similarly, in Homozygous for rs266729 polymorphism, the 4-week HPP in respect to baseline induced a significantly greater reduction in Glycemia levels than in both WT and Heterozygous. The Heterozygous for rs266729 polymorphism showed a significantly greater reduction in Triglycerides levels, than WT. Finally, the Heterozygous for rs17300539 polymorphism took advantage of a significantly greater reduction in weight than WT. Although at the univariate analysis the Heterozygous for rs62625753 polymorphism showed a greater advantage in adiponectin levels increase after the HPP, the multivariate regression analysis, after correction for confounding factors, showed a significant association between Δ adiponectin and WT polymorphism, suggesting the people with severe obesity WT for rs62625753 could have a greater advantage by the HPP in respect to the Heterozygous.

To the best of our knowledge, this is the first study that, at the same time, considered and correlated adiponectin levels, biochemical parameters and the *ADIPOQ* genetic background. Faraj et al. examined fasting adiponectin levels before and 15 months after bypass surgery in 50 people with severe obesity [45]. They found that the extent of weight loss after surgery was related to the pre-operative adiponectin concentrations. The lower pre-operative adiponectin levels predicted a greater percentage of reduction in body weight, and they were associated with higher increases in adiponectin levels. However, the Faraj study did not consider the genetic background [45]. The molecular mechanisms underlying the correlation between *ADIPOQ* SNPs and biochemical parameters modulation is still largely unknown; synergistic effects with polymorphisms in other gene involving in metabolic functions might intervene. Shin et al. [46] demonstrated that the SNP 276G>T of the ADIPOQ gene is associated with different responses of circulating adiponectin and insulin resistance to mild weight loss in overweight-obese patients, but they did not consider lipid profile. Nascimiento et al. found that IL-1β, IL-6, and TNF-α levels are associated with the *ADIPOQ* rs1501299 c.214 + 62G>T polymorphism, suggesting a relationship between the *ADIPOQ* and the inflammatory status [47]. 

Since polymorphisms in the promoter region of the *ADIPOQ* can regulate adiponectin expression, our data about SNPs in this region (i.e., rs17300539, rs266729) indicate an indirect regulatory role of adiponectin on lipid and/or glucose metabolism. Regarding the polymorphisms located in the exon 2, as well as in the intron region, further analyses of more SNPs in additional genes will be necessary to understand the cooperation of several genes in the regulation of metabolic parameters.

The strength of the study is that our cohort of severe people with severe obesity is constituted by large sample size in a controlled study. On the other hand, the limitation of the study is the short follow-up period and the absence of data about adverse outcomes.

## 5. Conclusions

Our study confirmed that a 4-week program consisting of physical exercise and diet in people with severe obesity results in an amelioration of both anthropometrical and biochemical parameters, together with a significant increase in adiponectin levels with specific regard to HMW and MMW oligomers. Importantly, we found that a specific genetic background in the *ADIPOQ* gene can predispose toward a more significant weight loss. Further studies are needed to deepen the effects of a combined program of diet and physical exercise and the genetic background in severe obesity.

## Figures and Tables

**Table 1:** General and biochemical parameters of the control group and the people with severe obesity at T0.

	Controls *mean ± SD*	People with Severe Obesity *mean ± SD*	*p* Value
Age (years)	40.84 ± 9.40	42.28 ± 13.81	0.390
Gender (F/M)	101/49	181/87	0.966
Weight (Kg)	67.08 ± 11.81	128.83 ± 23.31	**<0.0001**
BMI (Kg/m^2^)	23.46± 2.89	48.55 ± 7.60	**<0.0001**
Glucose (mg/dL)	81.38 ± 12.14	96.57 ± 34.80	**<0.0001**
Total cholesterol (mg/dL)	150.03 ± 41.83	183.44 ± 36.87	**<0.0001**
HDL cholesterol (mg/dL)	55.46 ± 16.37	41.15 ± 10.32	**<0.0001**
LDL cholesterol (mg/dL)	104.23 ± 31.97	119.28± 30.15	**0.001**
Triglycerides (mg/dL)	98.71 ± 38.88	146.28 ± 50.73	**<0.0001**
Fibrinogen (mg/dL)	274.67 ± 44.57	422.57 ± 104.85	**<0.0001**
Adiponectin (µg/mL)	29.89 ± 9.86	24.07 ± 6.67	**<0 0001**

F. female; M. male; BMI. Body Mass Index; HDL. High Density Lipoptrotein; LDL. Low Density Lipoprotein.

**Table 2:** Comparison of general and biochemical parameters of the people with severe obesity at baseline (T0) and at the end of a 4-week Hypocaloric diet and Physical exercise Program (HPP) (T1).

People with Severe Obesity	T0 *mean ± SD*	T1 *mean ± SD*	*p*-Value
Weight (Kg)	128.83 ± 23.31	120.49 ± 22.31	**<0.0001**
BMI (Kg/m^2^)	48.55 ± 7.60	45.46 ± 7.11	**<0.0001**
Glucose (mg/dL)	96.57 ± 34.80	86.62 ± 23.22	**<0.0001**
Fasting Insulin	12.43 ± 6.03	13.06 ± 7.50	0.085
HOMA-IR-	3.03 ± 2.04	2.95 ± 2.28	0.455
2-h glucose	107.78 ± 38.17	103.67 ± 22.33	**0.030**
Total cholesterol (mg/dL)	183.46 ± 36.93	164.10 ± 34.15	**<0.0001**
HDL cholesterol (mg/dL)	45.36 ± 13.44	40.82 ± 10.92	**<0.0001**
LDL cholesterol(mg/dL)	118.35 ± 31.89	96.99 ± 29.67	**<0.0001**
Triglycerides(mg/dL)	146.28 ± 50.73	128.76 ± 41.47	**<0.0001**
Fibrinogen (mg/dL).	418.32 ± 107.05	403.26 ± 101.71	**0.007**
VES	33.01 ± 25.05	26.67 ± 22.00	**<0.0001**
CRP	10.00 ± 10.72	7.76 ± 11.67	**0.002**
Adiponectin (µg/mL)	24.03 ± 6.63	24.81 ± 6.95	**<0.0001**

HOMA-IR. Homeostasis model assessment for Insulin Resistance; HDL. High Density Lipoprotein; LDL. Low Density Lipoprotein; VES. Velocity of Erythrocyte Sedimentation; CRP. C-Reactive Protein.

**Table 3:** Adiponectin considered polymorphisms in controls and people with severe obesity.

		Controls *n (%)*	People with Severe Obesity *n (%)*	*p* Value
**rs 266729 c.-11377 C > G**	Wt	111 (74)	183 (68.3)	0.217
	Hetero	31 (20.7)	75 (28.0)	
	Homo	8 (5.3)	10 (3.7)	
**rs 16861194 c.-11426 A > G**	Wt	141 (94)	240 (89.6)	0.053
	Hetero	9 (6.0)	18 (6.7)	
	Homo	0 (0.0)	10 (3.7)	
**rs 17300539 c.-11391 G > A**	Wt	115 (76.7)	230 (86.0)	**0.012**
	Hetero	35 (23.3)	36 (13.4)	
	Homo	0 (0.0) 1	2 (0.6)	
**rs60806105 c.-11156 insCA**	Wt	141 (94)	241 (89.9)	**0.018**
	Hetero	9 (6)	27 (10.1)	
	Homo	0 (0.0)	0 (0.0)	
**rs 2241766 c.45 T > G**	Wt	104 (69.3)	178 (66.4)	0.820
	Hetero	41 (27.3)	81 (30.2)	
	Homo	5 (3.3)	9 (3.4)	
**rs1501299 c.214+62 G > T**	Wt	103 (68.7)	138 (51.6)	**<0 0001**
	Hetero	42 (28.0)	107 (39.8)	
	Homo	5 (3.3)	23 (8.6)	
**rs62625753 c.268G > A**	Wt	148 (98.7)	264 (98.5)	0.896
	Hetero	2 (1.3)	4 (1.5)	
	Homo	0 (0.0)	0 (0.0)	
**rs 17366743 c.331T > C**	Wt	144 (96.0)	252 (94.0)	0.266
	Hetero	6 (4)	16 (6.0)	
	Homo	0 (0.0)	0 (0.0)	

**Table 4:** Differences in Δ values for the significant biochemical parameters on the basis of the main genotypes in people with severe obesity.

Variables	WT *n* (%)	Hetero *n* (%)	Homo *n* (%)	*p* Value
**rs266729 c.-11377C>G**	183 (68.3)	75 (28.0)	10 (3.7)	
ΔGlucose	8.36 ± 13.24 *	9.31 ± 11.94 *	42.70 ± 23.65	**<0.0001**
ΔTriglycerides	14.63 ± 19.16	24.77 ± 19.80 ^#^	23.90 ± 7.98	**<0.0001**
**rs17300539c.11391G>A**	230 (86.0)	36 (13.4)	2 (0.6)	
ΔWeight	7.78 ± 3.49	9.16 ± 3.52 ^$^	12.9 ± 4.4	**0.005**
**rs62625753 c.268G>A**	264 (98.5)	4 (1.5)	0 (0)	
Δtotal cholesterol	20.25 ± 15.41	−3.0 ± 1.73 ^@^	-	**0.010**
ΔLDL-cholesterol	24.46 ± 12.37	9.33 ± 4.04 ^!^	-	**0.036**
ΔAdiponectin	−1.06 ± 2.06	−4.48 ± 1.78 ^	-	**0.005**

Δ was calculated as difference between T0 minus T1. * Homo vs another group *p* < 0.0001; # WT vs Hetero *p* = 0.004; ^$^ WT vs Hetero *p* = 0.029; ^&^ Homo *vs* other group *p* = 0.002; ^%^ Hetero vs Homo. *p* = 0.045; ^@^ WT vs Hetero *p* = 0.010; **^!^** WT vs Hetero *p* = 0.036; ^ WT vs Hetero *p* = 0.005.

## Supplementary Materials

The following are available online at https://www.mdpi.com/2072-6643/11/9/2195/s1, Figure S1: Genomic DNA of adiponectin Table S1: Differences in Δ values for the biochemical parameters on the basis of the main genotypes in people with severe obesity, Table S2: Wild tipe alleles, Location and Hardy Weiber equilibrium p values of the considered polymorphisms.
