# Supplementary material for: Adiponectin Expression and Genotypes in Italian People with Severe Obesity Undergone a Hypocaloric Diet and Physical Exercise Program
Source: Nutrients. 2019 Sep 12;11(9):2195. doi: 10.3390/nu11092195 (PMC6769478; doi:10.3390/nu11092195)
Supplement: Supplementary file 1 [file nutrients-11-02195-s001.pdf]

nutrients MDPI

Supplementary Table S1. Differences in Δ values for the biochemical parameters on the basis of the main genotypes in people with severe obesity.

Variables	WT n (%)	Hetero n (%)	Homo n (%)	*p* value
**rs266729 c.-11377C>G**	183 (68.3)	75 (38.0)	10 (3.7)	
Δ Weight	8.37±3.54	7.63±3.51	8.84±3.909	.261
Δ Glucose	8.36±13.24[*]	9.31±11.94[*]	42.70±23.65	**<.000**
Δ Triglycerides	14.63±19.16	24.77±19.80[#]	23.90±7.98	**<.000**
Δ total cholesterol	19.39±15.73	21.37±16.80	28.90±5.67	.143
Δ LDL-cholesterol	23.07±13.74	26.51±13.88	31.30±7.86	.051
Δ Adiponectin	2.267±.168	2.074±.240	2.667±.843	.281
**rs16861194 c.-11426 A>G**	240 (89.6)	18 (6.7)	10 (3.7)	
Δ Weight	7.95±3.489	8.51±2.402	9.08±3.964	.496
Δ Glucose	9.14±13.700	9.67±9.84	14.90±14.41	.417
Δ Triglycerides	17.71±19.82	15.33±16.44	24.80±18.90	.458
Δ total cholesterol	20.41±15.99	17.67±15.21	22.30±14.88	.718
Δ LDL-cholesterol	24.15±14.01	23.56±10.85	30.20±10.68	.383
Δ Adiponectin	-1.12±1.96	-1.96±1.94	-1.18±1.95	.203
**rs17300539c.11391G>A**	230 (86.0)	36 (13.4)	2 (0.6)	
Δ Weight	7.78±3.49	9.16±3.52[$]	12.9±4.4	**.005**
Δ Glucose	8.79±12.46	13.06±19.13	17.50±21.92	.152
Δ Triglycerides	17.10±19.40	22.06±20.84	24.00±7.07	.335
Δ total cholesterol	20.36±15.94	19.64±15.97	25.50±2.12	.870
Δ LDL-cholesterol	23.99±13.55	26.28±15.18	29.00±2.83	.580
Δ Adiponectin	-1.16±2.20	-1.40±2.46	-1.30±2.54	.835
**rs60806105 c.-11156 insCA**	241 (89.9)	27 (10.1)	0 (0.0)	
Δ Weight	8.04±3.50	7.94±2.97	---	.889
Δ Glucose	9.23±12.88	10.22±18.75	---	.719
Δ Triglycerides	17.80±19.39	17.96±21.53	---	.968
Δ total cholesterol	20.15±15.97	21.63±15.05	---	.646
Δ LDL-cholesterol	23.98±13.67	27.52±14.06	---	.204
Δ Adiponectin	-1.24±2.22	-.70±2.35	---	.227
**rs2241766 c.45 T>G**	178 (66.4)	81 (30.2)	9 (3.4)	
Δ Weight	7.81±3.43	8.47±3.50	8.55±3.01	.325
Δ Glucose	8.00±11.86	11.69±16.15	14.22±16.69	.069
Δ Triglycerides	17.38±19.80	18.22±19.57	22.89±15.71	.696
Δ total cholesterol	20.45±15.82	19.41±16.21	25.33±13.91	.556
Δ LDL-cholesterol	24.01±13.98	24.46±13.60	29.67±9.11	.483
Δ Adiponectin	-1.18±2.33	-1.16±2.11	-1.57±1.33	.870
**rs1501299 c.214+62 G>T**	138 (51.6)	107 (39.8)	23 (8.6)	
Δ Weight	7.68±3.53	8.37±3.30	8.63±3.45	.209
Δ Glucose	7.55±10.91	10.88±16.00	12.92±14.75	.066
Δ Triglycerides	17.23±19.25	17.21±19.28	23.88±22.36	.284
Δ total cholesterol	19.28±15.78	22.30±15.98	17.42±15.40	.219
Δ LDL-cholesterol	24.12±12.91	26.88±17.75	24.04±13.83	.638
Δ Adiponectin	-1.26±2.11	-.91±2.37	-2.07±2.03	.057
**rs62625753 c.268G>A**	264 (98.5)	4 (1.5)	0 (0)	
Δ Weight	8.04±3.46	7.70±2.49	---	.848
Δ Glucose	9.21±13.53	17.25±14.25	---	.239

Δ Triglycerides	17.88±19.63	13.75±16.66	---	.676
Δ total cholesterol	20.25±15.41	-3.0±1.73[@]	---	**0.010**
Δ LDL-cholesterol	24.46±12.37	9.33±4.04[!]	---	**0.036**
Δ Adiponectin	-1.06±2.06	-4.48±1.78^	---	**0.005**
**rs 17366743 c.331T>C**	252 (94.0)	16 (6.0)	0 (0.0)	
Δ Weight	8.01±3.48	8.35±2.93	---	.701
Δ Glucose	9.12±13.43	12.69±15.28	---	.307
Δ Triglycerides	17.58±19.32	21.50±23.60	---	.439
Δ total cholesterol	20.32±15.78	20.00±17.67	---	.938
Δ LDL-cholesterol	24.11±13.66	27.88±14.70	---	.288
Δ Adiponectin	-1.16±2.24	-1.64±2.14	---	.410

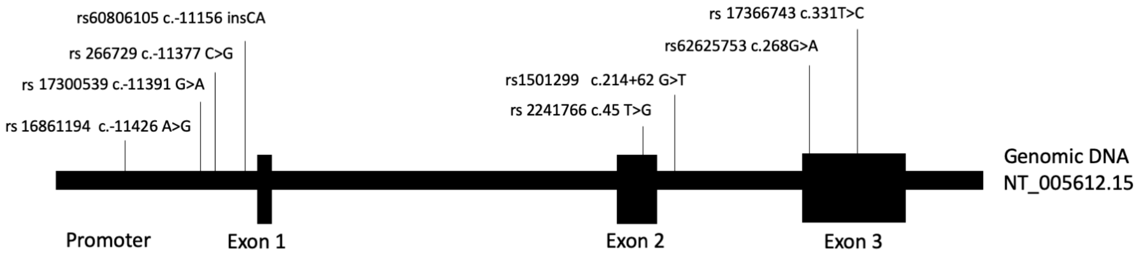

Supplementary Figure S1. Genomic DNA of adiponectin.

Supplementary Table S2. Wild tipe alleles, Location and Hardy Weiber equilibrium p values of the considered polymorphisms.

Considered Polymorphism	Wild Type Allele	Location	HWE *p* Value
**rs 266729 c.-11377 C>G**	CC	Promoter	0.697
**rs 16861194 c.-11426 A>G**	AA	Promoter	0.071
**rs 17300539 c.-11391 G>A**	GG	Promoter	0.921
**rs60806105 c.-11156 insCA**	No CA insertion	Promoter	0.594
**rs 2241766 c.45 T>G**	TT	Exon 2	0.988
**rs1501299 c.214+62 G>T**	GG	Intron 2	0.815
**rs62625753 c.268G>A**	GG	Exon 3	0.940
**rs 17366743 c.331T>C**	TT	Exon 3	0.757

HWE, Hardy Weinberg Equilibrium estimated by using the calculator on http://www.dr-petrek.eu/documents/HWE.xls.
